# Endovascular mechanical thrombectomy for the treatment of extensive postpartum cerebral venous sinus thrombosis: a case report

**DOI:** 10.3389/frph.2026.1925471

**Published:** 2026-10-02

**Authors:** Mariana Acosta Chiquillo, Rafael Leonardo Aragón Mendoza, Andrés Contreras, María Lucia Suárez Benítez, Víctor S. Rangel, Angela María Muñoz, Jorge L. Campo, Jose Alejandro Flórez Cardona

**Affiliations:** 1 Instituto de la Mujer, Hospital Universitario Mayor-Méderi, Bogotá, Colombia; 2 Universidad del Rosario, Bogotá, Colombia; 3 Clinical Investigation Group, Universidad del Rosario, Bogotá, Colombia

**Keywords:** cerebral venous thrombosis, endovascular procedures, puerperium, stroke, thrombectomy

## Abstract

**Background:**

Cerebral venous sinus thrombosis (CVST) is a rare but potentially life-threatening complication of pregnancy and the postpartum period. Although systemic anticoagulation remains the standard first-line treatment, evidence supporting endovascular mechanical thrombectomy in postpartum patients is limited.

**Case presentation:**

A 22-year-old woman presented 12 days after an uncomplicated vaginal delivery with progressive headache and vomiting. Neuroimaging confirmed extensive CVST involving the superior sagittal, cortical, left transverse, and sigmoid sinuses, extending to the left jugular bulb. An initially suspected bilateral parieto-occipital convexity subarachnoid hemorrhage was subsequently ruled out following further imaging review. Despite prompt anticoagulation with low-molecular-weight heparin, she developed rapid neurological deterioration with decreased level of consciousness. Following multidisciplinary evaluation, endovascular mechanical thrombectomy was performed using aspiration and stent-retriever techniques, achieving successful venous recanalization. The procedure was complicated by transient vasospasm, which resolved after intra-arterial milrinone administration. An extensive evaluation excluded inherited and acquired thrombophilia, autoimmune disease, malignancy, and infection, identifying the postpartum period as the most likely predisposing factor. The patient was discharged 12 days after the intervention with therapeutic anticoagulation and anti-seizure medication. At six-month follow-up, she demonstrated complete neurological recovery without residual deficits.

**Conclusion:**

This case highlights that endovascular mechanical thrombectomy may represent a valuable therapeutic option in carefully selected postpartum patients with extensive CVST who develop progressive neurological deterioration despite anticoagulation. Although current evidence remains limited, this report supports the role of a multidisciplinary approach and timely endovascular intervention in achieving favorable neurological outcomes.

## Introduction

Cerebral venous sinus thrombosis (CVST) is a rare cerebrovascular disorder, with an estimated annual incidence of approximately 5 cases per million population, accounting for 0.5%–1.0% of all strokes (1, 2). Its etiology is multifactorial and involves both inherited risk factors, such as thrombophilias, and acquired conditions, including surgery, trauma, pregnancy, and the postpartum period (3–5).

The incidence of CVST is more than threefold higher in women than in men, a difference that has been attributed to female-specific risk factors (1, 6). This disparity is particularly relevant in women's health, especially during pregnancy and the postpartum period, as CVST represents a potentially life-threatening complication, with reported mortality rates ranging from 3% to 15%, most deaths occurring within the first 30 days after symptom onset (3, 7).

Systemic anticoagulation remains the first-line treatment for CVST and is associated with favorable outcomes when initiated promptly. However, a subset of patients with progressive neurological deterioration or intracerebral hemorrhage may benefit from adjunctive invasive therapies, including endovascular interventions such as catheter-directed thrombolysis and mechanical thrombectomy, as well as decompressive neurosurgery (3, 8). The aim of this report is to describe the case of a woman in the late postpartum period who developed extensive cerebral venous sinus thrombosis and was successfully treated with endovascular mechanical thrombectomy, resulting in complete neurological recovery without residual deficits at follow-up.

The study was conducted in accordance with the principles of the Declaration of Helsinki. Written informed consent was obtained from the patient for publication of this case report and the accompanying images. Ethical approval was granted by the Human Research Ethics Committee of Hospital Universitario Mayor–Méderi (Approval No. CEISH-2026042). The report was prepared in accordance with the CARE guidelines for case reports.

## Case presentation

A 22-year-old woman with no significant past medical history presented to the emergency department 12 days after an uncomplicated spontaneous vaginal delivery. She had received postpartum contraception with depot medroxyprogesterone acetate (150 mg). She reported a 36-h history of sudden-onset, progressively worsening diffuse headache, exacerbated by Valsalva maneuvers and associated with multiple episodes of emesis containing gastric contents. On admission, she was hemodynamically stable, with a Glasgow Coma Scale score of 15 and no focal neurological deficits on examination.

Laboratory investigations, including a complete blood count, metabolic panel, renal and liver function tests, and coagulation profile, were within normal limits. Given the presence of headache with red flag features, a non-contrast computed tomography (CT) scan of the brain was performed, demonstrating marked hyperdensity of the superior sagittal, left transverse, and straight sinuses, highly suggestive of acute cerebral venous sinus thrombosis. Based on this presumptive diagnosis, therapeutic anticoagulation with low-molecular-weight heparin (LMWH; enoxaparin 1 mg/kg subcutaneously every 12 h) was initiated. However, approximately 13 h after anticoagulation initiation, the patient developed acute neurological deterioration. Subsequent magnetic resonance venography confirmed extensive dural venous sinus thrombosis involving the entire superior sagittal sinus, cortical veins, left transverse and sigmoid sinuses, and the left jugular bulb. These findings were initially associated with suspected bilateral parieto-occipital convexity subarachnoid hemorrhage; however, this diagnosis was subsequently ruled out following further imaging review (Figures 1, 2).

**Figure 1:**
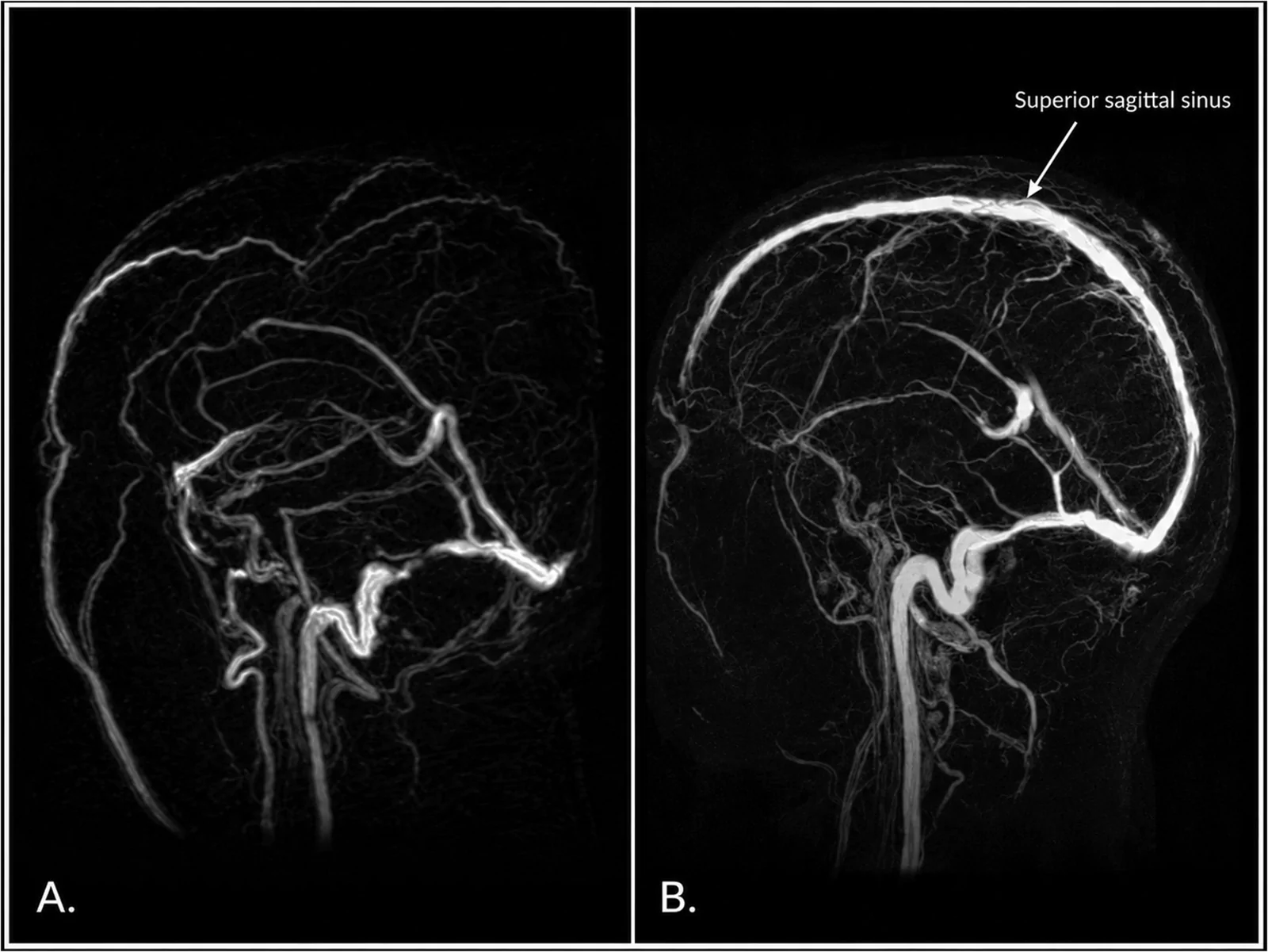
Contrast-enhanced MR brain venography before and after endovascular mechanical thrombectomy. **(A)** Pretreatment MR venogram demonstrating absent opacification of the superior sagittal sinus, consistent with cerebral venous sinus thrombosis. **(B)** Follow-up MR venogram demonstrating restoration of contrast opacification and patency of the superior sagittal sinus after endovascular treatment. The arrow indicates the recanalized superior sagittal sinus.

**Figure 2:**
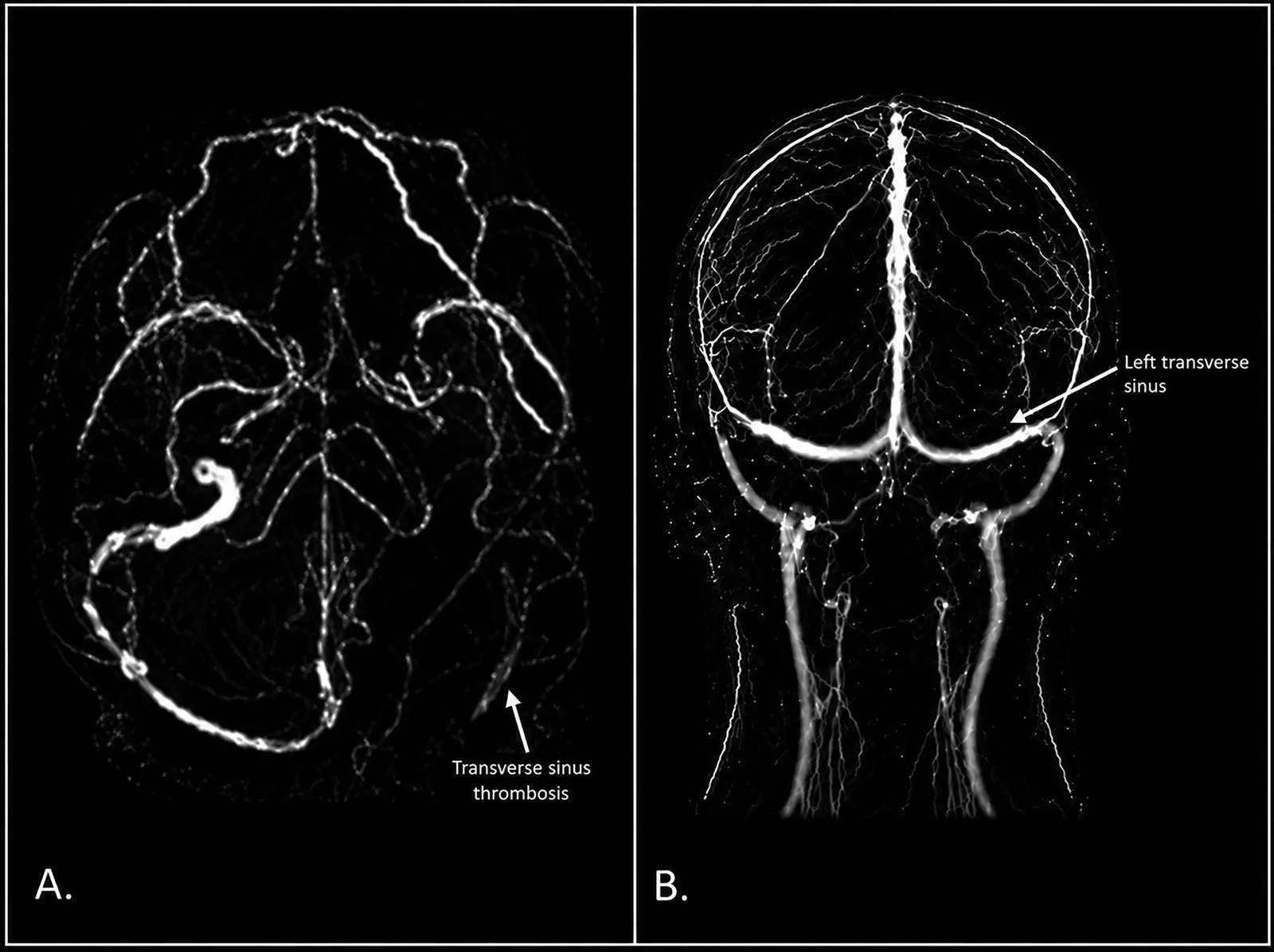
Contrast-enhanced MR brain venography before and after endovascular mechanical thrombectomy. **(A)** Pretreatment inferior maximum-intensity projection showing absence of contrast opacification of the left transverse-sigmoid venous system, consistent with cerebral venous sinus thrombosis (arrow). **(B)** Follow-up coronal maximum-intensity projection demonstrating restoration of contrast opacification of the left transverse and sigmoid sinuses after endovascular mechanical thrombectomy. The arrow identifies the recanalized left transverse sinus.

Following evaluation by a multidisciplinary team comprising Neurology, Neurosurgery, and Interventional Neuroradiology, the initial management strategy consisted of continued medical treatment with close monitoring in the intensive care unit. However, during hospitalization, the patient experienced rapid neurological deterioration, progressing to a stuporous state with a Glasgow Coma Scale score of 12, characterized by eye opening to verbal stimuli, confused verbal responses, and purposeful motor localization to pain, accompanied by the development of bilateral mydriasis. An urgent follow-up non-contrast brain CT scan showed no significant interval changes compared with the previous examination. However, given the marked discrepancy between stable imaging findings and the patient's rapid neurological deterioration, the multidisciplinary team decided to proceed with endovascular mechanical thrombectomy. This decision was based on the presence of high-risk features for clinical deterioration, including the postpartum state and extensive cerebral venous thrombosis with complete superior sagittal sinus occlusion. Importantly, neuroimaging did not demonstrate significant parenchymal lesions, space-occupying lesions, or mass effect; therefore, the Neurosurgical team did not recommend decompressive surgery. The neurological deterioration was considered primarily related to persistent venous congestion and impaired venous outflow secondary to extensive sinus thrombosis, supporting endovascular thrombectomy as a rescue strategy targeting the underlying pathophysiological mechanism. Due to the urgency of the clinical situation and the need for immediate intervention, neither electroencephalography nor invasive intracranial pressure monitoring was performed prior to neurointervention.

The procedure was performed under general anesthesia via right femoral arterial and right femoral venous access. A diagnostic cerebral angiogram was initially obtained using a 5-French vertebral catheter advanced over a hydrophilic guidewire, confirming complete thrombosis of the superior sagittal sinus as well as the left transverse and sigmoid sinuses (Figure 3). A Neuron 088 guide catheter was subsequently advanced into the right internal jugular vein. Using a coaxial system, a RED 68 reperfusion catheter was navigated to the superior sagittal sinus, where mechanical thrombectomy was performed. The RED 68 catheter was then repositioned within the left transverse sinus. Through a coaxial system consisting of a Rebar 18 microcatheter supported by an Avigo microwire, the left sigmoid sinus was successfully catheterized. A 6 × 40 mm Solitaire X stent retriever was subsequently deployed, and mechanical thrombectomy of the left transverse and sigmoid sinuses was performed. Final angiography demonstrated successful recanalization of the left transverse and sigmoid sinuses (Figure 4). The procedure was complicated by catheter-induced vasospasm involving the C1 segment of the internal carotid artery during diagnostic catheterization. Targeted intra-arterial milrinone was administered as rescue therapy to reverse the focal vasospasm, restore cerebral blood flow, and reduce the risk of downstream ischemic injury through its local vasodilatory effect. The total procedure duration was 87 min, resulting in a Qureshi Recanalization Scale score of IIb.

**Figure 3:**
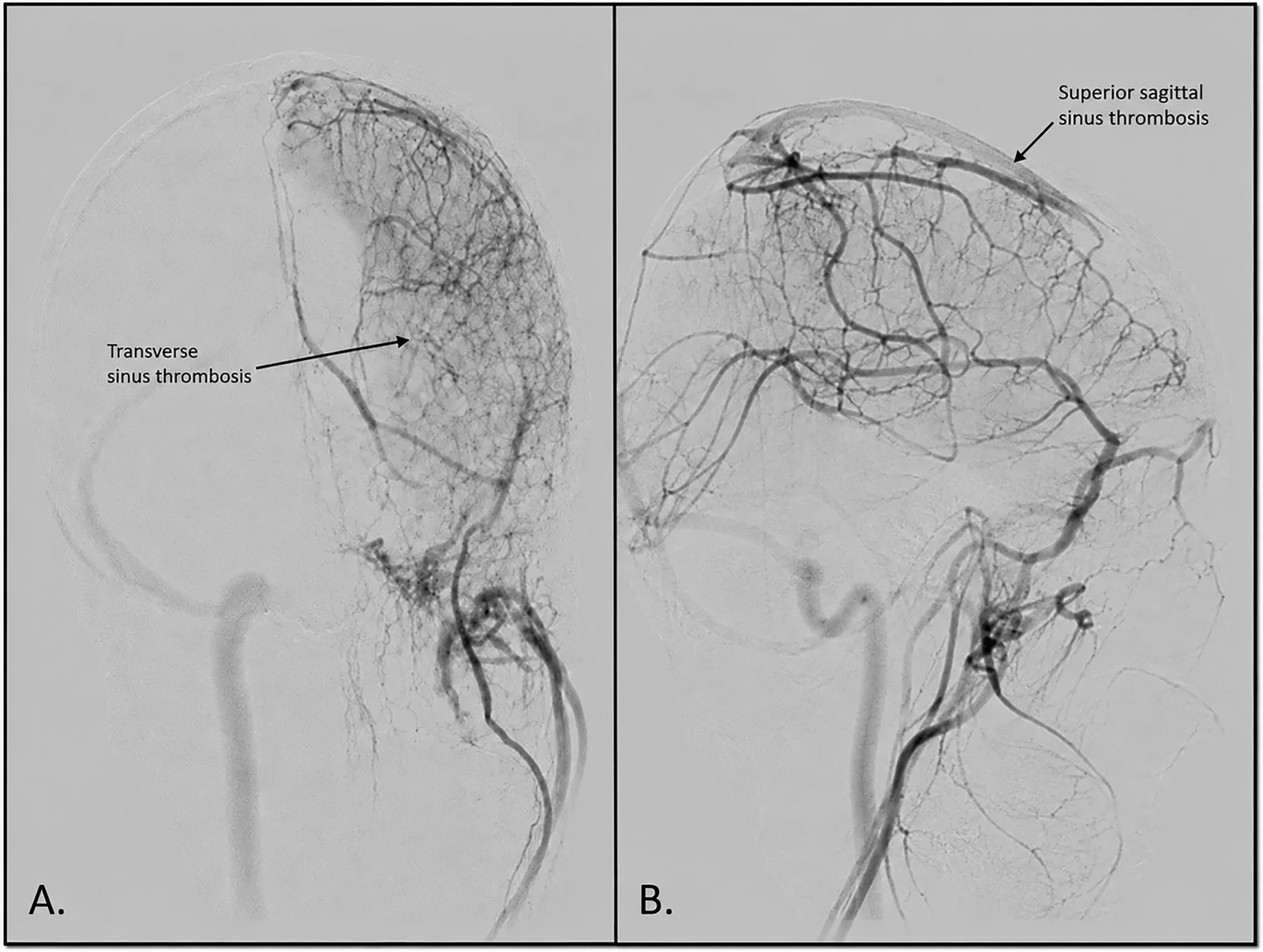
Cerebral angiography demonstrating thrombosis of the superior sagittal sinus, left transverse sinus, and left sigmoid sinus. **(A)** Anteroposterior projection showing absence of opacification of the left transverse sinus (arrow). **(B)** Lateral projection demonstrating absence of opacification of the superior sagittal sinus (arrows), associated with prominent cortical venous collateralization, consistent with impaired cerebral venous drainage.

**Figure 4:**
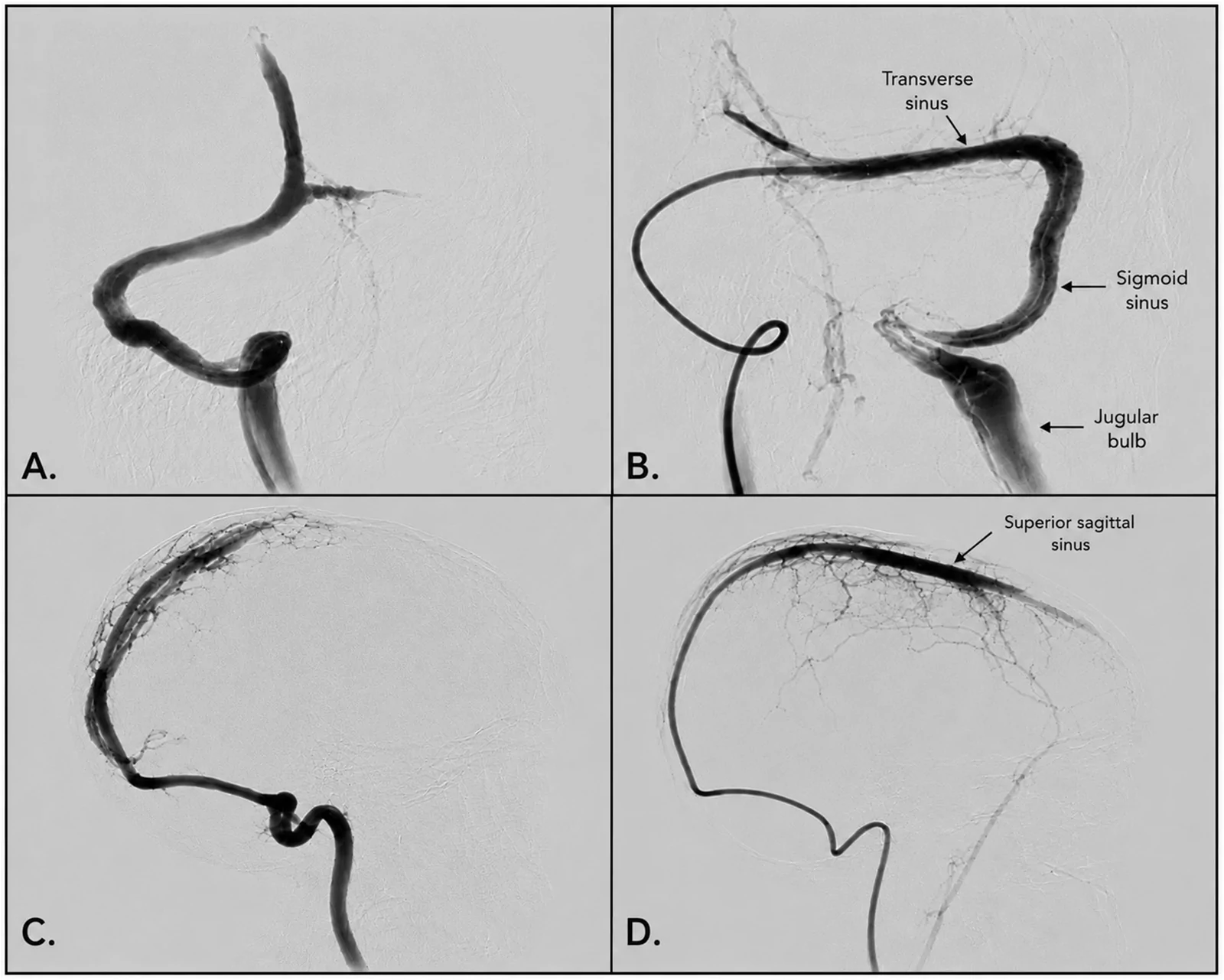
Cerebral venography before and after endovascular mechanical thrombectomy. **(A)** Pretreatment venogram showing absent opacification of the left transverse and sigmoid sinuses, consistent with cerebral venous sinus thrombosis. **(B)** Final venogram demonstrating restoration of antegrade venous drainage through the recanalized left transverse sinus, sigmoid sinus, and jugular bulb. Arrows indicate the recanalized venous drainage pathway. **(C)** Pretreatment venogram demonstrating absent contrast opacification of the superior sagittal sinus, consistent with cerebral venous sinus thrombosis. **(D)** Final venogram demonstrating restoration of contrast opacification and antegrade venous drainage through the recanalized superior sagittal sinus. The arrow identifies the superior sagittal sinus.

Two days after the procedure, vasopressor support and invasive mechanical ventilation were successfully discontinued. However, the patient remained somnolent without further neurological deterioration. Follow-up brain computed tomography and CT angiography excluded parenchymal hemorrhage but demonstrated residual thrombosis involving the posterior two-thirds of the superior sagittal sinus, the left parietal cortical veins, and the left transverse and sigmoid sinuses. These findings were interpreted as residual thrombus burden without indication for repeat endovascular intervention. Given the fluctuating level of consciousness and clinical suspicion of seizure activity, antiseizure treatment was initiated with benzodiazepines and a loading dose of levetiracetam, followed by progressive neurological improvement, including recovery of pupillary reactivity, increased alertness, interaction with the environment, and the ability to follow simple commands. Continuous video-electroencephalography subsequently demonstrated mild encephalopathy without epileptiform activity. However, considering the initial suspicion of ictal activity and the presence of multiple risk factors for seizures, including extensive cerebral venous thrombosis and acute lacunar infarcts within the territory of the medullary veins, lacosamide was temporarily added during the acute phase.

Dual antiseizure therapy with levetiracetam (1 g every 12 h) and intravenous lacosamide (200 mg every 12 h) was considered a temporary therapeutic strategy in the setting of a high-risk clinical scenario rather than prophylactic treatment. With favorable neurological recovery and no further seizure-like episodes, lacosamide was discontinued, and the patient was maintained on levetiracetam monotherapy. The therapeutic approach was guided by clinical suspicion of acute symptomatic seizures and subsequently tailored according to the patient's neurological evolution.

To investigate the underlying etiology, a comprehensive diagnostic workup was performed. Malignancy screening with contrast-enhanced computed tomography of the neck, chest, abdomen, and pelvis revealed no evidence of neoplastic disease. Autoimmune testing, including antinuclear antibodies, anti-double-stranded DNA antibodies, and complement levels, was unremarkable. A comprehensive thrombophilia evaluation, including both inherited and acquired causes, yielded negative results, and active infection was excluded. Collectively, these findings identified the postpartum period as the most likely predisposing factor for the development of cerebral venous sinus thrombosis.

Following clinical improvement, the patient was discharged 12 days after the endovascular intervention. Therapeutic anticoagulation with low-molecular-weight heparin (enoxaparin, 1 mg/kg administered subcutaneously every 12 h) was prescribed for six months. Anti-seizure therapy was continued with oral levetiracetam (1,000 mg every 12 h). In addition, residual headache, characterized by burning and lancinating pain with localized scalp allodynia, was clinically classified as neuropathic and managed with oral paracetamol/caffeine (500/65 mg every 8 h) and pregabalin (50 mg every 12 h).

At the 6-month follow-up, the patient showed favorable clinical progress, with a score of 1 on the modified Rankin Scale, attributable to minor intermittent headaches. The patient reported progressive clinical improvement, characterized by a marked reduction in the frequency and intensity of headaches, allowing her to resume her normal daily activities. No seizures, focal neurological symptoms, or residual neurological deficits were identified. Cognitive and functional recovery was satisfactory, with no limitations in daily activities or social reintegration. Magnetic resonance imaging and venography at the 3-month follow-up showed no evidence of new dural venous thrombosis. The transverse sinuses remained patent on angiographic evaluation, with only residual stenosis of the superior sagittal sinus. These findings indicate favorable venous evolution after treatment.

Overall, the experience was perceived positively, with particular appreciation for the multidisciplinary care received throughout hospitalization and rehabilitation. Anticoagulation with low-molecular-weight heparin (enoxaparin) was continued, and levetiracetam was maintained at 1,000 mg orally every 12 h. The patient remains under neurological follow-up, pending reassessment of the need for continued antiseizure therapy and evaluation for gradual withdrawal of levetiracetam based on clinical evolution.

## Discussion

CVST is an uncommon cerebrovascular disorder with multiple established risk factors (1, 6). However, pregnancy and, particularly, the postpartum period represent times of markedly increased susceptibility (9). Cohort studies have demonstrated that the risk of venous thromboembolism increases progressively throughout pregnancy, reaching its highest level during the first three weeks postpartum. Compared with nonpregnant women, the risk is approximately sixfold higher during the third trimester of pregnancy (3).

The diagnosis of postpartum CVST remains clinically challenging because its most common presenting symptom, headache, may mimic other conditions frequently encountered during the postpartum period, including post-dural puncture headache following neuraxial anesthesia and hypertensive disorders of pregnancy, particularly eclampsia, which commonly presents initially with headache followed by seizures, thereby contributing to delays in diagnosis (1). In this context, maintaining a high index of clinical suspicion is essential to establish a timely diagnosis and initiate prompt treatment. The superior sagittal sinus is the most commonly affected site, involved in up to 62% of cases, likely owing to its extensive cortical venous drainage (10). This is followed by the transverse and sigmoid sinuses, which are affected in approximately 45% of patients and are frequently associated with temporoparietal venous infarctions (3).

In selected patients who develop neurological deterioration, expanding intracerebral hemorrhage, or progression of venous infarction despite therapeutic anticoagulation, adjunctive invasive therapies may be considered (4). Among these, endovascular treatment offers the potential for early recanalization and restoration of physiological cerebral venous drainage (3, 6). Mechanical thrombectomy, in particular, may offer advantages over catheter-directed thrombolysis by achieving rapid and direct recanalization without the need for fibrinolytic agents, potentially reducing the risk of hemorrhagic expansion in patients with concomitant intracerebral hemorrhage (3). In patients with intracranial hypertension secondary to space-occupying lesions, decompressive craniectomy represents an additional therapeutic option aimed at reducing intracranial pressure (2, 3).

Although the literature extensively documents cases of CVST occurring during pregnancy and the postpartum period that respond favorably to systemic anticoagulation, evidence supporting the use of endovascular interventions remains limited. In the published cases, the primary indication for endovascular mechanical thrombectomy has been progressive neurological deterioration despite therapeutic anticoagulation, which was also the indication for intervention in our patient. While pregnancy and the postpartum period are well-established risk factors because of the physiological hypercoagulable state, several reported cases have also involved additional prothrombotic conditions. For example, Chow et al. described a patient with positive antiphospholipid antibodies (4), whereas Serna et al. reported a case associated with heterozygous factor V Leiden mutation, elevated factor VIII levels, and increased lipoprotein A concentrations (11). In contrast, our patient underwent a comprehensive evaluation for both inherited and acquired thrombophilias, as well as an extensive workup to exclude other secondary causes, all of which yielded negative results. Consequently, the postpartum period was considered the most likely predisposing factor for the development of CVST.

Li et al. evaluated the safety and efficacy of mechanical thrombectomy combined with local thrombolysis as a therapeutic strategy for patients with severe CVST. Their study included 52 patients, among whom eight were either pregnant or in the postpartum period, demonstrating that this treatment approach is a safe and effective therapeutic option for patients with severe disease or those who do not respond adequately to systemic anticoagulation (12). In our case, mechanical thrombectomy was performed because of progressive neurological deterioration despite therapeutic anticoagulation.

Current evidence regarding endovascular therapy (EVT) for CVST remains limited. The TO-ACT randomized clinical trial did not demonstrate a significant improvement in functional outcomes compared with standard medical therapy alone (13). However, postpartum women were markedly underrepresented in the study, with only three postpartum patients included, thereby limiting the applicability of its findings to obstetric populations. Recent systematic reviews evaluating EVT have reported favorable recanalization rates, good functional outcomes, low mortality, and low rates of postprocedural hemorrhage in patients who failed systemic anticoagulation therapy. However, these reviews did not include specific analyses of pregnant or postpartum populations, and the available evidence for obstetric patients therefore remains limited. Similarly, the systematic review by Bücke et al. concluded that the current evidence does not support the routine use of EVT for CVST. However, EVT may represent a reasonable therapeutic option in carefully selected patients at high risk of poor outcomes. The authors also emphasized the need for further studies to better define patient selection, optimal endovascular techniques, and the timing of treatment initiation (14). Notably, only one study included in this systematic review reported specific data on pregnant patients, highlighting the limited evidence available for obstetric populations.

Among patients with CVST who receive timely treatment, the prognosis is generally favorable, with approximately 75% achieving complete functional recovery (6). Nevertheless, a subset of patients may experience death, persistent physical disability, or long-term neuropsychiatric sequelae, including anxiety and depression, underscoring the importance of a multidisciplinary approach throughout the recovery process (6). Following mechanical thrombectomy, the time to complete neurological recovery is variable, ranging from five weeks to six months in published reports (1, 6). Consistent with these findings, our patient achieved complete neurological recovery without residual deficits at follow-up.

The optimal long-term anticoagulation strategy following CVST in the postpartum period remains an area of ongoing discussion. Although recent clinical trials have demonstrated that direct oral anticoagulants are an effective alternative for selected patients with CVST, evidence supporting their use during breastfeeding remains limited (5, 15). Consequently, LMWH continues to represent a preferred therapeutic option in lactating women because of its well-established safety profile and extensive clinical experience. Furthermore, contemporary obstetric guidelines for the management of pregnancy-associated venous thromboembolism recommend therapeutic LMWH for postpartum women requiring prolonged anticoagulation, supporting its use in clinical scenarios such as the one presented in this report (9, 16, 17).

Regarding the use of anti-seizure medication, therapeutic decisions in patients with CVST should be contextualized according to their clinical presentation, underlying structural brain abnormalities, and neurological evolution. In our patient, anti-seizure therapy was initiated in the setting of the initial neurological presentation and the substantial structural and anatomical risk for seizures associated with CVST. Although subsequent video-EEG monitoring demonstrated mild encephalopathy without active epileptiform discharges, this finding represented a specific monitoring window and was therefore interpreted within the broader clinical context rather than in isolation. Given the persistence of underlying seizure risk, anti-seizure medication was continued during the acute phase. This approach is consistent with evidence indicating an increased risk of acute symptomatic and subsequent seizures in patients with CVST presenting with focal neurological deficits or parenchymal brain involvement (18). Thus, decisions regarding the initiation, continuation, and eventual discontinuation of anti-seizure medication should be individualized according to the patient's clinical presentation, structural brain injury, neurological course, and overall seizure risk.

Postpartum contraceptive counseling is an essential component of the long-term management of women with CVST, as it aims not only to reduce the risk of recurrent thrombotic events but also to prevent unintended pregnancies that may increase the risk of adverse maternal outcomes (3, 5). In the present case, the patient received depot medroxyprogesterone acetate (DMPA) for contraception. Previous studies have shown that initiation of DMPA during the immediate postpartum period is associated with a low absolute incidence but an increased relative risk of venous thromboembolism compared with nonuse of hormonal contraception (19). Therefore, contraceptive counseling should be individualized according to each patient's baseline thrombotic risk. Estrogen-containing contraceptives should be avoided, and DMPA should be used with caution in women at moderate to high risk of venous thromboembolism (20).

This report has several limitations. First, as a single-case report, its findings cannot be generalized to the broader population of patients with postpartum CVST. Additionally, the inherent nature of a case report precludes establishing causal relationships or drawing conclusions regarding the comparative effectiveness of the therapeutic approach. Furthermore, some technical details of the endovascular thrombectomy procedure were not available, limiting a more comprehensive description and reproducibility of the intervention. Despite these limitations, this case contributes to the limited evidence regarding the multidisciplinary management of postpartum CVST requiring endovascular treatment.

## Conclusion

Cerebral venous sinus thrombosis is a rare but potentially life-threatening complication of the postpartum period that requires a high index of clinical suspicion. Prompt diagnosis and early initiation of therapeutic anticoagulation are essential to reduce morbidity and mortality and to prevent permanent neurological sequelae.

Although systemic anticoagulation remains the cornerstone of treatment, this case illustrates that endovascular mechanical thrombectomy may represent a valuable therapeutic option in carefully selected patients with progressive neurological deterioration, intracranial hemorrhage, or failure to respond to anticoagulation. However, the available evidence remains limited, and further studies are needed to better define the indications, safety, and efficacy of this intervention in the postpartum population.

## Data Availability

The original contributions presented in the study are included in the article/Supplementary Material, further inquiries can be directed to the corresponding author.
